# Influence of Probiotics on the Animal Gut Microbiota and Their Impact on the Bioavailability of Toxic Agents: An Opinion Paper

**DOI:** 10.3389/fnut.2022.870162

**Published:** 2022-04-18

**Authors:** Adrián Hernández-Mendoza, Aarón F. González-Córdova, Marcel Martínez-Porchas

**Affiliations:** Centro de Investigación en Alimentación y Desarrollo, A.C. Coordinación de Tecnología de Alimentos de Origen Animal, Carretera Gustavo Enrique Astiazarán Rosas, Hermosillo, Mexico

**Keywords:** probiotics, gut microbiota, xenobiotic absorption, bioremediation, dysbiosis, toxic agent

## Introduction

Humans and animals coexist with microbes and have a vital relationship with many. Most of these microbes are vastly contained in the gastrointestinal tract, defined as the gut microbiota, a critical “symbiont” for the development and survival of humans and animals due to its multiple functionalities favoring the host (1).

The gut microbiota is unique in each individual in terms of microbial structure and function; however, stress, social interactions, diet, nutrient, pharmacologic factors, and many other stimuli, including biological agents like antibiotics and xenobiotics, play critical roles in the modulation of gut microbial composition (2, 3). In this last regard, environmental xenobiotics are chemicals to which an organism is exposed that are extrinsic to its normal metabolism (4); some of these are substances of known toxicity, substances known to be inert, and substances whose toxicity or inertness remains to be established. Toxic xenobiotics include natural (e.g., mycotoxins) as well as synthetic compounds such as pesticides, drugs, additives, heavy metals, food additives. From the mechanistic view, they may come into contact with the gastrointestinal system causing physiological and/or anatomical abnormalities by interfering in the host biological processes. These also can affect the structure and functions of the host's gut microbiota, disrupting the synergistic relationship between the host and its microbiota with subsequent physiological, metabolic, and immune implications (3, 5).

Over the last decade, extensive evidence has shown that probiotics, defined as “live microorganisms that, when administered in adequate amounts, confer a health benefit on the host” (6), are capable of surviving at the gastrointestinal tract after oral intake and reduce the bioaccessibility of xenobiotics by acting as binding agents (7). However, studying the effect of probiotics on shaping the gut microbiota to modulate/modify the dietary xenobiotic lifetime and bioavailability has not been as extensive. Hence, in this document, we support the hypothesis that probiotics can shape the functions of the gut microbiota to withstand and metabolize toxic agents.

## Xenobiotics and the Gut Microbiota

**Figure 1:**
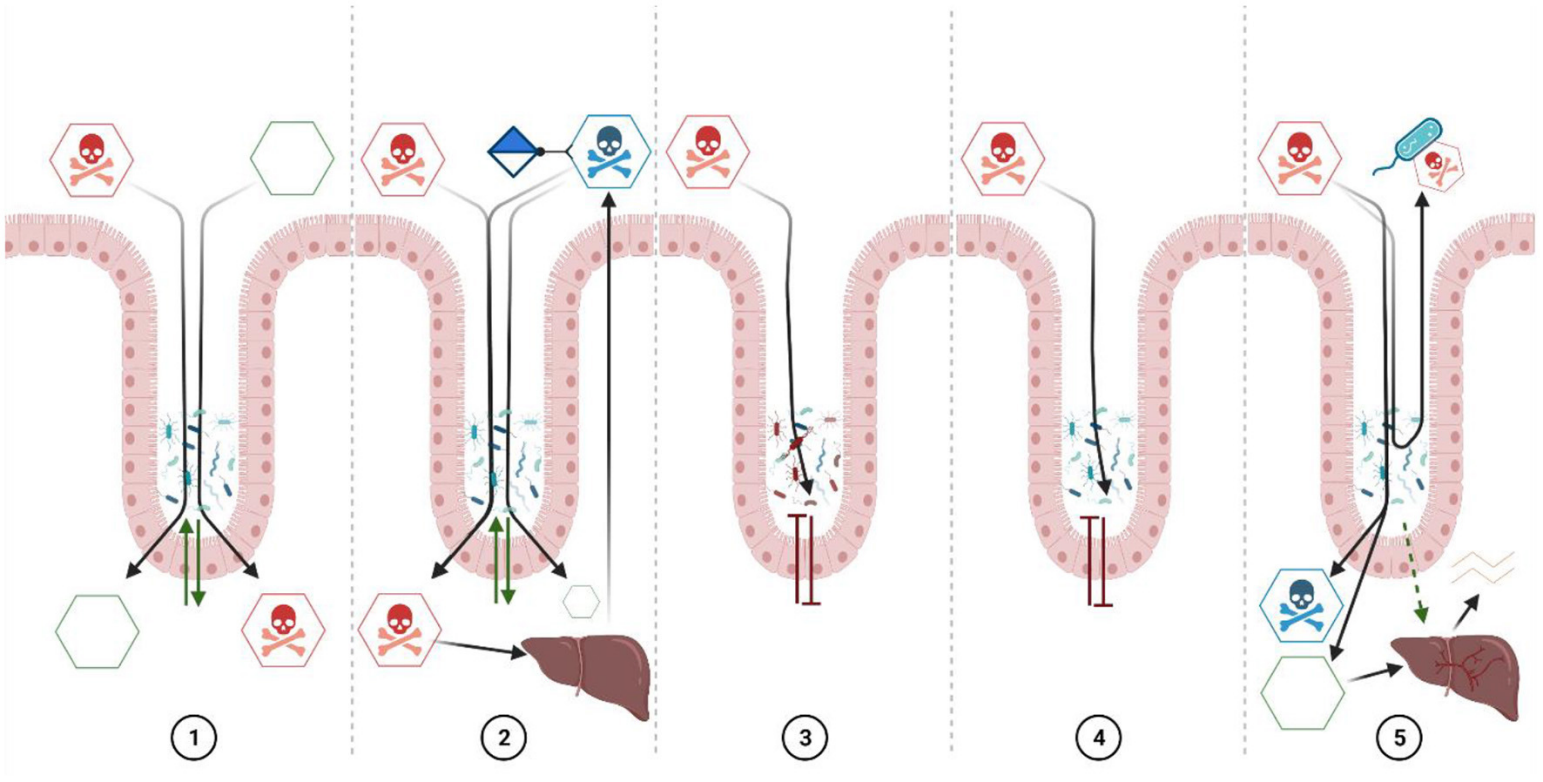
Xenobiotic interaction with the intestinal microbiota. 1. Xenobiotics not affected by oral cavity, stomach, and small intestine digestion, or poorly adsorbed, are displaced to the distal small intestine and cecum by peristalsis, and the microbiota residing in the large intestine may directly metabolize those partitioned through the intestinal wall from the blood. 2. Due to the non-polar nature of most xenobiotics, they are easily absorbed by the intestinal tract and subsequently transported through the bloodstream to the liver where some xenobiotics are oxidized, forming conjugates (with sulfates, glucuronic acid or glutathione) that can be excreted by the bile and metabolized by the microbiota which, in turn, transforms these conjugates into non-polar molecular conjugates of low molecular weight with low toxicity that are adsorbed again; however, the microbiota can also deconjugate these compounds and “release” the xenobiotic again. 3. Some xenobiotics can affect the gut microbiota, either by affecting specific taxonomic groups or favoring others. The function of the intestinal microbiota is affected and, with it, the symbiotic relationship with the host. 4. Although some xenobiotics do not affect the abundance of gut microbiota members, they can affect the function of the microbiota. Some changes may occur in the activity of endogenous metabolites or the general metabolic capacity of the microbiota, also affecting the symbiotic relationship with the host. 5. The intestinal microbiota could induce an enzymatic response at the liver tissue level, increasing the host's detoxification capacity. Also, the microbiota can produce active and inactive variants of the xenobiotic, which can be metabolized by liver tissue. Finally, some microbiota bacteria inactivate the xenobiotic by direct interaction (binding) on the cell surface, decreasing the adsorption of the xenobiotic. However, there could be multiple additional mechanisms that have yet to be elucidated. Based on Claus et al. (8) with modifications and insertions. Created with BioRender.com.

Xenobiotics can gain access into living organisms by multiple routes; however, most xenobiotics enter the human body through the gastrointestinal tract. Evidence has determined several scenarios after incorporating a xenobiotic (8–10); here, we list at least five of them: 1 the gut microbiota can directly metabolize the xenobiotic, 2 the gut microbiota can metabolize the xenobiotic after conjugation with the liver (via enteropathic cycling) or, if not metabolized, 3 the xenobiotic may induce dysbiosis, 4 interfere with the enzymatic activity of the gut microbiota, 5 inactivate the xenobiotic by direct interaction (binding) on the cell surface and others (Figure 1).

The gut microbiota harbors a diversity of about >1,000 microbial species providing an extensive set of metabolic functions (11). For instance, gut microbes can metabolize dozens of drugs via acetylation, deacetylation, deconjugation, deglycosylation, dehydroxylation, demethylation, denitration, reduction, N-oxide bounds cleavage, proteolysis, etcetera (12). Furthermore, it has been reported that some gut microorganisms can directly alter the chemical structures of xenobiotics; however, the microbial strains and enzymes (and their associated genes) involved in the metabolism of xenobiotics are still poorly understood (13). In this regard, enzyme families such as β-glucuronidases, β-lyases, azoreductases, nitroreductases, and sulfatases have been detected in the gut microbiota (14–16). For instance, gut microbes can reduce a considerable diversity of functional groups such as α,β-unsaturated carboxylic acid derivatives, alkenes, nitro, N-oxide, azo, and sulfoxide groups, decreasing the polarity of compounds with the consequent charge alteration, hybridization, and electrophilicity, their toxicities and lifetimes in the body (13).

Overall, the activity of the gut microbiota reduces the final absorption of xenobiotics by hosts and their bioavailability (17, 18); however, if xenobiotics breach the intestinal epithelium, the gut microbiota can trigger the host's immune response. In this regard, members of the gut microbiota can synthesize and release metabolites capable of modulating the host immune response, altering hepatic gene expression for dealing with xenobiotics, competing for enzymes and drug transporters, and acting as intermediates (18, 19). Withal, as stated, xenobiotics may not always be metabolized, causing dysbiosis by inhibiting, promoting, or eliminating gut microbiota members; therefore, such microbiota members must be protected, and probiotics could play a critical protective role.

Several factors can influence gut microbiome xenobiotic metabolism, including host genome, age, geography, diet, gender, hormonal status, circadian rhythms, and others (9). However, interventions aiming to modulate the gastrointestinal tract's microbial ecology and favoring groups with these functions may provide new insights regarding microbially mediated xenobiotics-transformations for human health benefits.

## Use of Probiotics as Regulators of Microbial Interaction Networks in the Gastrointestinal Microbiota: A Hypothetical Approach

Advances in sequencing technologies have revealed the association between dysbiosis (the loss of beneficial gut species, absence of microbial diversity and/or pathogen domination of the gut) and a wide range of human diseases. For instance, studies have evidenced that the imbalance of intestinal microbiota and changes in microbiota composition is closely related to immune-mediated neuropathies, such as Guillain-barre syndrome (GBS), which is characterized by elevated levels of Th1 and Th17 proinflammatory cytokines, reduction of anti-inflammatory Th2 and Th3 cytokines, along with quantitative and qualitative defects of regulatory T cells (Treg), suggesting the crucial roles of these important mediators for the onset and progression of GBS (20). Considering that the microbiota modulates host immune responses and affects the production of cytokines, it has been hypothesized that clinical symptoms of GBS may be improved by regulating the imbalance of intestinal microbiota. In this regard, studies have shown that probiotics (e.g., *Lactobacillus helveticus* R0052, *Bifidobacterium infantis*) may improve GBS immune balance by reducing the most common bacteria associated with GBS, namely *Campylobacter jejuni, Escherichia*, and *Coprococcus* (21). Besides, probiotic *Bifidobacterium infantis* administration improves the imbalance of Th cell subsets (Th1, Th2, Th17) and promotes Treg cells' levels, thereby ameliorating GBS symptoms (22, 23).

In the same context, gut dysbiosis is closely associated with colorectal cancer (CRC) development. At the genus and species level, *Bacteroides fragilis, Enterococcus faecalis, Streptococcus bovis, Escherichia coli, Fusobacterium* spp., *Ruminococcus* spp., and *Peptostreptococcus* spp., are suspected to be involved in colorectal carcinogenesis (24). Despite the exact mechanisms involving these bacteria in colorectal pathogenesis are still unclear, *in vitro* and *in vivo* studies have evidenced the key role of enterotoxigenic strains, whose respective toxin produced (e.g., colibactin, polyamines) affect pathways leading to increased cell proliferation, the release of proinflammatory effectors, and DNA damage (25). Moreover, considering that bacterial adherence is often a prerequisite step to tumor promotion, virulence factors, such as the *afa, eae*, and *Fap2* adhesins, may induce CRC by selectively binding to E-cadherin and activating the β-catenin signaling pathway, thus inducing oncogenic and inflammatory responses. In this same context, aggregations of microbial communities encased in a polymeric matrix (biofilms) enhanced colonic epithelial permeability, which facilitates bacterial antigen translocation and promotes pro-carcinogenic tissue inflammation. Besides, ROS-producing bacteria can activate pro-carcinogenic factors and CRC-promoting pathways (26).

On the other hand, coincidence in reducing the abundance of butyrate-producing bacteria and in the carbohydrate-degradation genes responsible for SCFA production has been reported in CRC patients. Furthermore, fat-mediated alterations of the gut microbiota link bile acid metabolism to CRC since changes in the gut microbiota may promote an altered overall bile acid pool, which activates or restricts intestinal and hepatic cross-signaling of the bile acid receptor, farnesoid X receptor (FXR), which plays a crucial role in regulating intestinal cell proliferation and carcinogenesis (26, 27).

Based on the aforementioned, gut microbiota dysbiosis, bacterial virulence factors, and microbial-derived metabolism play vital roles in colorectal carcinogenesis; hence, it has been suggested that probiotics may be used to shape the gut microbiota to reduce the risk of colorectal cancer pathogenesis. In this sense, the beneficial effects of probiotics in CRC have been demonstrated *in vitro, in vivo*, and in preclinical trials, particularly for species of *Lactobacillus, Enterococcus*, and *Bifidobacterium*. However, the beneficial impact of probiotic supplementation depends on the strain, dosage, duration of the intervention, host physiology, and other food supplements. Proposed mechanisms of action include modulation or enhancement of immune function, the release of specific bioactive metabolites (e.g., antimicrobial peptides, organic acids, enzymes), improvement of barrier function, epithelial structure and metabolism, and even the microRNAs expression profile in the context of the microbiota composition (28, 29).

Despite the above research progresses, using probiotics for shaping and favoring a specific microbial community that tolerates and can metabolize toxic agents is a rare approach in the area, mainly because understanding the interaction networks between microorganisms to manipulate them is a complex challenge. However, some clues and partial evidence could provide a moderate insight; for instance, the survival of some bacteria even under adverse conditions may be favored by the presence of others. For example, Narisawa et al. (30) demonstrated that resistant bacteria could mediate the coexistence of excluding groups of bacteria like antibiotic-sensitive and antibiotic-producing bacteria. Even though this evidence was obtained from biofilms, the principle could apply to the gut microbiota, which is also a kind of biofilm. In this sense, we arise the question: could some probiotics favor the tolerance of essential but sensitive members of the gut microbiota, even under the presence of toxic agents? Considering that some probiotic mechanisms include components secretions, metabolic activity, and cell to cell interactions that could directly or indirectly favor sensitive members, the theoretical answer is affirmative.

Interaction network models could provide deeper insights to search for bacteria or groups that favor gut microbiota members' persistence with specific functions. In practice, the modulation of commensal microbiota by the consumption of transiting probiotics has been associated with antimicrobial compounds produced by probiotics or indirectly by modulating the immune system and gut barrier function (31); however, these are single links in a vast interaction network. For example, Kato et al. (32) monitored the network relationship of four different bacteria strains capable of degrading cellulose (strains S, F, 3, and 5). Briefly, results revealed that the survival of strain S depended on the effects of strain 5, but strain 5 was susceptible to strain 3 that showed bactericidal activity against this strain. However, strain F quenched the growth of strain 3 and favored the persistence of strain 5. These direct and indirect network relationships, including suppressive effects, resulted in balance, maintaining the colony's coexistence and functioning. Probiotics can be used in this way to favor networks that assist bacteria with specific capabilities, in this case, metabolize toxic agents. For instance, finding co-occurrence and core networks involving microbial specimens capable of metabolizing xenobiotics may provide evidence for designing single-strain or multispecies consortia formulations favoring such networks. Herein, the design of synthetic microbial consortia for gut microbiota modulation is an approach to consider (33).

Whether prebiotics can support the persistence of specific bacterial groups, sometimes these may not be as effective under adverse conditions involving toxic agents harming important microbes. However, the presence of bacteria mediating the persistence of others through the modification of interaction networks may be used; in other words, it is essential to gain insight into the principles driving bacteria-bacteria interactions because bacterial community networks determine central microbiota functions. In addition, the chemical communications between microbes through quorum sensing must be incorporated in this approach because predicting and manipulating these processes could favor the probability of success. However, knowing in deep these networks is a monumental challenge that must be solved by using metadata and robust computer systems that allow predicting the behavior of microbial communities in the presence of a specific probiotic. Also, using Oligo-Mouse-Microbiota 12 (OMM12) synthetic bacterial community as model research to understand microbiota interactions could provide relevant information (34). In this regard, OMM12 is a known community of 12 mouse intestinal bacteria used for microbiome research in gnotobiotic mice (35).

Finally, understanding the impact of intestinal microbiota on toxic agents' metabolism and how probiotics can be used from the different approaches to favor these functions could be a step forward in probiotics science.

## Author Contributions

AH-M and MM-P: article conceptualization, writing, and review. AG-C: writing and review. All authors contributed to the article and approved the submitted version.

## Conflict of Interest

The authors declare that the research was conducted in the absence of any commercial or financial relationships that could be construed as a potential conflict of interest.

## Publisher's Note

All claims expressed in this article are solely those of the authors and do not necessarily represent those of their affiliated organizations, or those of the publisher, the editors and the reviewers. Any product that may be evaluated in this article, or claim that may be made by its manufacturer, is not guaranteed or endorsed by the publisher.
